# The development of a GPR44 targeting radioligand [^11^C]AZ12204657 for in vivo assessment of beta cell mass

**DOI:** 10.1186/s13550-018-0465-6

**Published:** 2018-12-27

**Authors:** Mahabuba Jahan, Peter Johnström, Ram K. Selvaraju, Marie Svedberg, Maria Sörhede Winzell, Jenny Bernström, Lee Kingston, Magnus Schou, Zhisheng Jia, Stanko Skrtic, Lars Johansson, Olle Korsgren, Lars Farde, Christer Halldin, Olof Eriksson

**Affiliations:** 1 Department of Clinical Neuroscience, Center for Psychiatric Research, Karolinska Institutet, Karolinska University Hospital, SE-171 76 Stockholm, Sweden; 2 PET Science Centre, Precision Medicine and Genomics, IMED Biotech Unit, AstraZeneca, Karolinska Institutet, Stockholm, Sweden; 3 Science for Life Laboratory, Department of Medicinal Chemistry, Uppsala University, Uppsala, Sweden; 4 Bioscience, Cardiovascular Renal and Metabolism, IMED Biotech Unit, AstraZeneca, Gothenburg, Sweden; 5 Discovery Biology, Discovery Sciences, IMED Biotech Unit, AstraZeneca, Gothenburg, Sweden; 6 Early Chemical Development, Pharmaceutical Sciences, IMED Biotech Unit, AstraZeneca, Gothenburg, Sweden; 7 Innovation Strategies & External Liaison, Pharmaceutical Technology & Development, AstraZeneca, Gothenburg, Sweden; 8 GMED Diabetes, Global Medicines Development, AstraZeneca, Gothenburg, Sweden; 9 Department of Immunology, Genetics and Pathology, Division of Immunology, Uppsala University, Uppsala, Sweden; 10 Lee Kong Chian School of Medicine, Nanyang Technological University, Singapore, Singapore; 11 Present address: Antaros Medical, Mölndal, Sweden

**Keywords:** G-protein-coupled receptor 44 (GPR44), Beta cell imaging, Islet imaging, Beta cell mass, Diabetes

## Abstract

**Background:**

The G-protein-coupled receptor 44 (GPR44) is a beta cell-restricted target that may serve as a marker for beta cell mass (BCM) given the development of a suitable PET ligand.

**Methods:**

The binding characteristics of the selected candidate, AZ12204657, at human GPR44 were determined using in vitro ligand binding assays. AZ12204657 was radiolabeled using ^11^C- or ^3^H-labeled methyl iodide ([^11^C/^3^H]CH_3_I) in one step, and the conversion of [^11^C/^3^H]CH_3_I to the radiolabeled product [^11^C/^3^H]AZ12204657 was quantitative. The specificity of radioligand binding to GPR44 and the selectivity for beta cells were evaluated by in vitro binding studies on pancreatic sections from human and non-human primates as well as on homogenates from endocrine and exocrine pancreatic compartments.

**Results:**

The radiochemical purity of the resulting radioligand [^11^C]AZ12204657 was > 98%, with high molar activity (MA), 1351 ± 575 GBq/μmol (*n* = 18). The radiochemical purity of [^3^H]AZ12204657 was > 99% with MA of 2 GBq/μmol. Pancreatic binding of [^11^C/^3^H]AZ12204657 was co-localized with insulin-positive islets of Langerhans in non-diabetic individuals and individuals with type 2 diabetes (T2D). The binding of [^11^C]AZ12204657 to GPR44 was > 10 times higher in islet homogenates compared to exocrine homogenates. In human islets of Langerhans GPR44 was co-expressed with insulin, but not glucagon as assessed by co-staining and confocal microscopy.

**Conclusion:**

We radiolabeled [^11^C]AZ12204657, a potential PET radioligand for the beta cell-restricted protein GPR44. In vitro evaluation demonstrated that [^3^H]AZ12204657 and [^11^C]AZ12204657 selectively target pancreatic beta cells. [^11^C]AZ12204657 has promising properties as a marker for human BCM.

## Introduction

Currently, no validated biomarkers for assessment of beta cell mass (BCM) exist. Accurate longitudinal imaging biomarkers reflecting pancreatic BCM are anticipated to foster progress in development of therapies, for type 1 and type 2 diabetes (T1D/ T2D) by for the first time enabling change in BCM as a clinical endpoint. This is of paramount importance for assessments of novel therapies aimed at preserving residual BCM, or even restore the pancreatic BCM to normal levels (i.e., a possible diabetes cure). Such a tool would promote the understanding of the natural development of the beta cells in the healthy human pancreas during the life span. Additionally, it would assist in unraveling the etiology of T1D/T2D by potentially filling critical gaps in current knowledge regarding the change in BCM during the course of the disease [1].

The G-protein-coupled receptor 44 (GPR44), also designated as the prostaglandin D2 (PGD_2_) receptor 2, DP2, or CRTh2, was recently identified in the pancreas as beta cell restricted in a proteomics screening effort [2]. Transcriptomic studies of pancreatic compartments corroborated the proteomic results, showing substantially higher GPR44 mRNA in the islets of Langerhans compared to exocrine tissue [3]. Additionally, GPR44 is located to the outer cell membrane and therefore accessible by potential imaging agents in the circulation [3].

Small molecule GPR44 antagonists, mainly indolic, hydroquinolinyl, and arylacetic acid derivatives, have previously entered clinical phase testing for indications in asthma and allergic rhinitis, due to the high expression on pro-inflammatory cells such as eosinophils, Th2 and basophils [4–6]. GPR44 tritiated antagonist AZD3825 with suitable tracer properties was generated as a part of a molecular library by AstraZeneca. [^3^H]AZD3825 bound with high potency to beta cells in a GPR44 mediated manner, measuring approximately 50 times higher GPR44 receptor density in beta cells compared to exocrine homogenates [3]. Furthermore, [^11^C]AZ12204657, structurally similar to AZD3825, exhibited islet specific targeting in pancreas in vivo in pigs and non-human primates [7]. GPR44 thus fulfills the strict requirements set for a putative molecular target for non-invasive tracking of pancreatic BCM [1].

The current study describes the selection, radiolabeling, and in vitro evaluation of positron emission tomography (PET) GPR44 radioligand [^11^C]AZ12204657.

## Materials and methods

### Radiochemistry

The precursors for labeling disodium (S)-2-(4-chloro-2-(2-chloro-4-sulfinatophenoxy)phenoxy) propanoate and (S)-2-(4-chloro-2-(2-chloro-4-(methylsulfonyl)phenoxy)-6-iodophenoxy) propanoic acid, the reference compound (S)-2-(4-chloro-2-[2-chloro-4-(methylsulfonyl)phenoxy]phenoxy) propanoic acid (AZ12204657), and the GPR44 antagonist AZD3825 used for blocking studies were obtained from AstraZeneca AB, Sweden. All other chemicals were purchased from commercial sources, analytically graded, and used without any further purification.

**Fig. 1:**
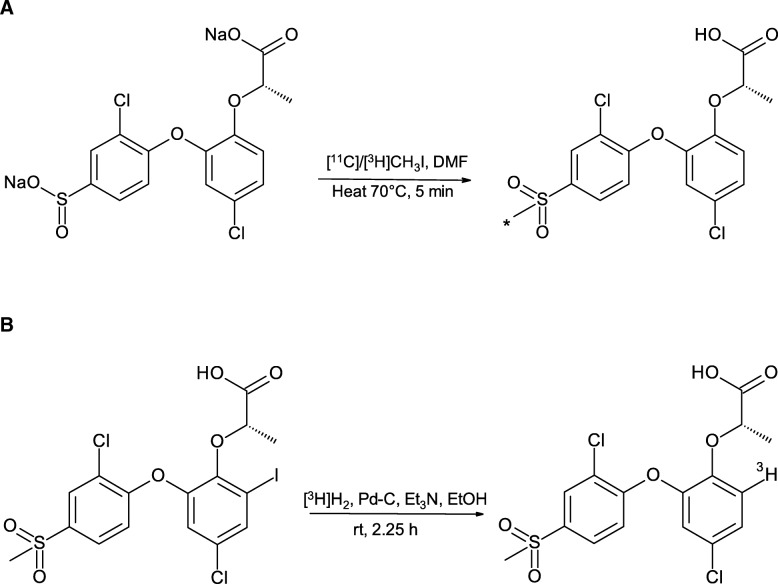
**a** Radiolabeling of AZ12204657 using [^11^C/^3^H]CH_3_I (position of label [*]). **b** Radiolabelling of AZ12204657 using [^3^H]H_2_

For in vitro studies, AZ12204657 was labeled with tritium (Fig. 1).

### Production of [^11^C] methyl iodide ([^11^C]CH_3_I)

[^11^C] Methane ([^11^C]CH_4_) was produced in-target via the ^14^N(p,α)^11^C reaction on nitrogen with 10% hydrogen, with 16.4 MeV protons using a GEMS PET trace cyclotron (GE, Uppsala, Sweden). Typically, the target gas was irradiated for 20–30 min with a beam intensity of 35 μA. Carbon-11 labeled methyl iodide, [^11^C]CH_3_I, was produced according to previously published method [8]. In short, [^11^C]CH_4_ was released from the target and isolated with liquid nitrogen followed by the release of [^11^C]CH_4_ by heating with pressurized air. Then [^11^C]CH_4_ was mixed with vapors from iodine crystals followed by a radical iodination reaction in a closed recirculation system to produce [^11^C]CH_3_I. The formed [^11^C]CH_3_I was collected and released to the reaction vessel. Radiomethylation, purification, and formulation were performed using a computer-controlled automated system (Scansys, Denmark).

### Radiosynthesis of [^11^C]AZ12204657

#### Radiochemistry

**Fig. 2:**
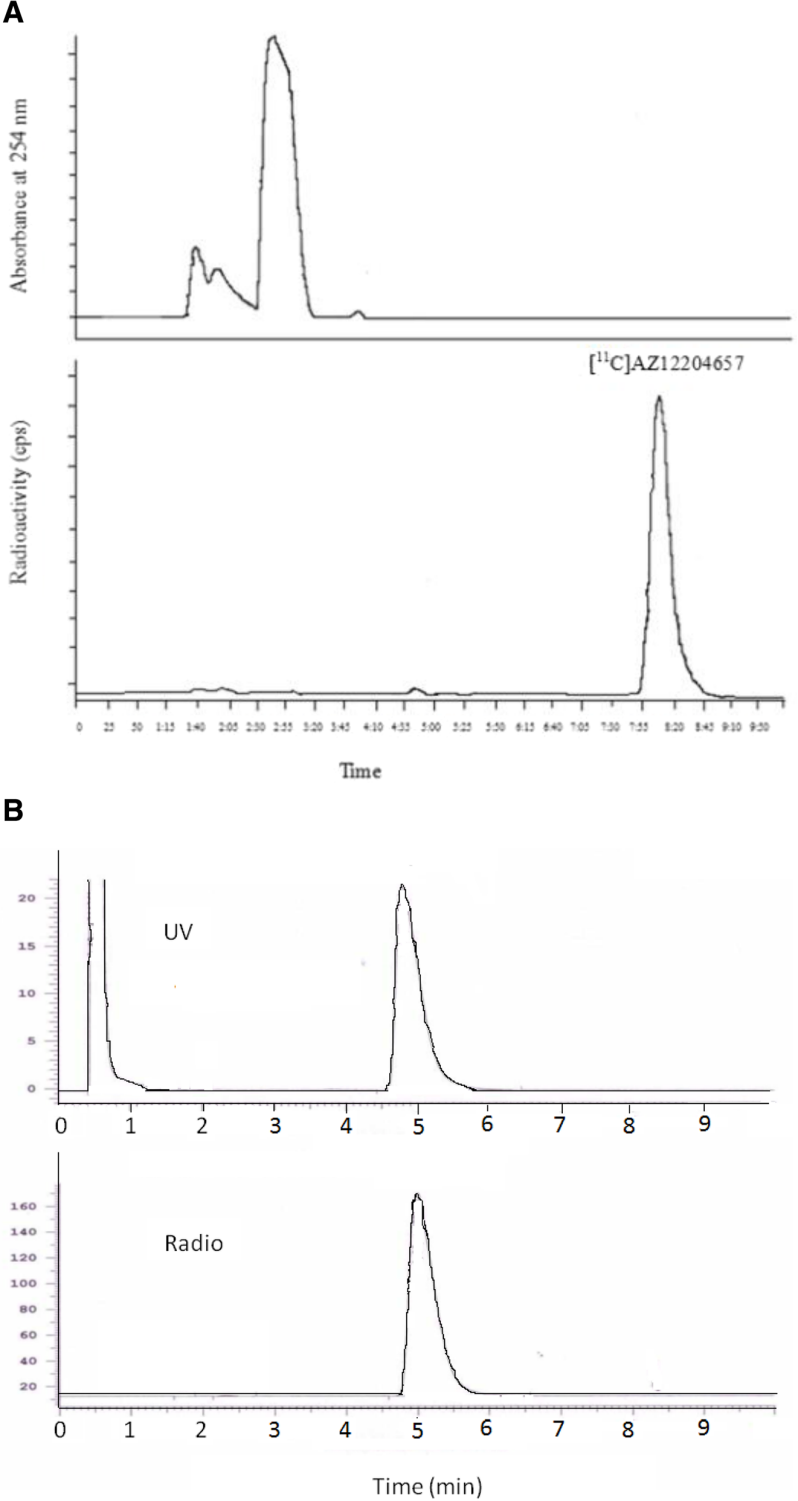
**a** HPLC Chromatograms of the semi-preparative purification, *R*_t_ ([^11^CAZ12204657) = 7–9 min. The lower trace shows radioactivity response, and the upper trace, absorbance response. The mobile phase system is CH_3_CN/0.1 M AF/sodium-l-ascorbate (35/65/0.5, *v/v/w*) at a flow rate of 4 mL/min. **b** The analytical HPLC chromatogram of co-injection of standard and radiolabeled [^11^C]AZ12204657; *R*_t_ = 4–6 min. The mobile phase system is CH_3_CN/0.1 M AF/sodium-l-Ascorbate (25/75/0.5, *v/v/w*) at a flow rate of 3 mL/min

Carbon-11 labeled AZ12204657 was obtained by trapping [^11^C]CH_3_I at room temperature (RT) in a reaction vessel containing disodium (S)-2-(4-chloro-2-(2-chloro-4-sulfinatophenoxy)phenoxy) propanoate (1.0–2.0 mg, 2.48–4.95 μmol) in dimethylformamide (DMF, 300 μL) (Fig. 1a). After the end of trapping, the reaction mixture was heated at 70 °C for 5 min. The reaction mixture was diluted with sterile water (500 μL) before injecting to the built-in high-performance liquid chromatography (HPLC) system for the purification of the radiolabeled compound. The HPLC system consisted of a semi-preparative reverse phase (RP) ACE column (C18, 10 × 250 mm, 5 μm particle size) and a Merck Hitachi UV detector (λ = 254 nm) (VWR, International, Stockholm, Sweden) in series with a GM-tube (Carroll-Ramsey, Berkley, CA, USA) used for radioactivity detection. The product was eluted with mobile phase of 35% acetonitrile (MeCN) in ammonium formate (AF, 0.1 M) containing sodium-l-ascorbate (500 mg/L) with a flow rate of 4 mL/min which gave a radioactive fraction corresponding to pure [^11^C]AZ12204657 (Fig. 2a). The collected fraction from HPLC was evaporated to dryness and re-formulated in 6–8 mL phosphate-buffered saline; PBS (pH 7.4).

The same radiosynthesis was explored using solid phase extraction (SPE) cartridge after semi-prep HPLC purification of the crude reaction mixture to skip evaporation of the mobile phase at high temperature (70–80 °C) to avoid radiolysis. The HPLC collected product fraction was diluted with 50 mL of water and loaded on a SEP-PAK-C18 cartridge (Waters). The SEP-PAK-C18 cartridge was pre-conditioned with 10 mL of EtOH (99.9%) and 10 mL of water prior to use. The trapped product, ([^11^C]AZ12204657), in the SEP-PAK-C18 cartridge was washed with water (10 mL) and eluted with EtOH (1 mL) in to PBS (9 mL). When using this method, no sodium-l-ascorbate needed to be added in the HPLC mobile phase, since no radiolysis was observed during SPE isolation.

The pH of the final formulated product was in the ranged of 6.5–7.5, and the product was sterile filtered through a Millipore Millex®GV filter unit (0.22 μm) before further use.

#### Quality control (QC)

The radiochemical purity, identity and stability of the radioligand [^11^C]AZ12204657 was identified using an analytical HPLC system which included an Eclips XDB RP column (Agilent, C18, 4.6 × 150 mm, 5 μm particle size), Merck-Hitatchi L-7100 Pump, L-7400 UV detector, and GM-tube for radioactivity detection (VWR International). A wavelength of 254 nm and a mobile phase system of 25% MeCN in AF (0.1 M) at flow rate of 3 mL/min was used for the analysis. The identity and the purity of [^11^C]AZ12204657 was confirmed by co-injection with unlabeled reference standard of AZ12204657 (Fig. 2b). The stability of the radiolabeled product, [^11^C]AZ12204657, was tested at different time intervals, 30, 60, 90, and 120 min after the synthesis using radio-HPLC.

#### Liquid chromatography-mass spectrometry (LC-MS/MS) analysis

LC-MS/MS analysis of the formulated product, [^11^C]AZ12204657, and of the reference standard, AZ12204657, was performed using a Waters Acquity™ ultra performance LC system connected with a Micromass premier™ Quadrupole time of flight (TOF) mass spectrometer (Waters, Milford, MA, USA). LC was performed using a Waters Acquity UPLC™ BEH column (C18, 2.1 × 50 mm, 1.7 μm particle size) kept at 50 °C. The mobile phase consisted of 0.1% formic acid in water (A) and 0.1% formic acid in MeCN (B). Samples were analyzed using a linear gradient (0 to 100% B in 5 min) at a flow rate of 0.5 mL/min. The MS was operated in electrospray ionization (+ESI) mode, with the following settings: capillary voltage 3.0 kV, cone voltage 35 V, source temperature 100 °C, dissolvation temperature 400 °C, and collision energy 20 eV. The formulated product solution was analyzed after radioactive decay without further dilution.

#### Molar activity determination

The molar activity (MA) of [^11^C]AZ12204657 was measured using an analytical HPLC system from Merck-Hitachi (VWR International) with D-6200A pump, L-4000 UV detector, and D-6000 interface. An Eclips XDB RP column (Agilent, C18, 4.6 × 150 mm, 5 μm particle size) was used with 25% MeCN in AF (0.1 M) at flow rate of 3 mL/min.

### Radiosynthesis of [^3^H]AZ12204657

AZ12204657 was labeled with tritium in two positions using ^3^H-methylation and palladium-catalyzed tritium-halogen exchange respectively.

#### (S)-2-(4-chloro-2-[2-chloro-4-([^3^H]methylsulfonyl)phenoxy]phenoxy) propanoic acid ([4-^3^H-methyl]-AZ12204657) (Fig. 1a)

[^3^H] Methyl Iodide ([^3^H]CH_3_I) was purchased from American Radiolabeled Chemicals (St. Louis, MO, USA), and the radiosynthesis was performed following the same procedure described for ^11^C-labeling (Fig. 1a). [^3^H]CH_3_I was added in the reaction vessel containing disodium (S)-2-(4-chloro-2-(2-chloro-4-sulfinatophenoxy)phenoxy) propanoate (1.0–2.0 mg, 2.48–4.95 μmol) in DMF (300 μL) at RT, and the reaction mixture was heated at 70 °C for 5 min. [4-^3^H-methyl]-AZ12204657 was purified and analyzed by HPLC (Merck Hitachi), equipped with UV detector and radiodetector (Packard). A semi-preparative RP column (ACE C18, 10 × 250 mm, 5 μm particle size) was used with an UV detector set at 254 nm for the purification of crude [4-^3^H-methyl]-AZ12204657 using 30:70 [MeCN:0.1 M AF] mobile phase composition at flow rate 5 mL/min. The solvent from the collected HPLC fraction was removed by evaporation under vacuum below 40 °C and afforded pure [4-^3^H-methyl]-AZ12204657. The isolated product was formulated with 70% EtOH in water.

The radiochemical purity, identity, and the stability of [4-^3^H-methyl]-AZ12204657 was identified using analytical HPLC system which included an ACE RP column (C18, 4.6 × 150 mm, 5 μm particle size), Merck-Hitatchi L-7100 Pump, L-7400 UV detector and a radiodetector (Packard). A wavelength of 254 nm and a mobile phase system of 30% MeCN in AF (0.1 M) at flow rate of 2 mL/min was used for the analysis. The identity and the purity of [4-^3^H-methyl]-AZ12204657 was confirmed by co-injection with unlabeled reference standard of AZ12204657.

The molar activity of [4-^3^H-methyl]-AZ12204657 was measured using analytical HPLC system from Merck-Hitachi (VWR International) with L-7100 Pump, L-7400 UV detector, and a radiodetector (Packard). An ACE RP column (C18, 4.6 × 150 mm, 5 μm particle size) was used with 30% MeCN in AF (0.1 M) at flow rate of 2 mL/min. MA was calibrated for UV absorbance (λ = 254 nm) response per mass of ligand and calculated as the radioactivity of the radioligand (GBq) divided by the amount of associated carrier substance (μmol). Each sample was analyzed two times and compared to a reference standard analyzed two times.

The obtained molar activity of [4-^3^H-methyl]-AZ12204657 was 2 GBq/μmol, and the radiochemical purity was > 99.9% up to 1 week after radiosynthesis when stored at − 18 °C.

#### (S)-2-(4-chloro-2-(2-chloro-4-(methylsulfonyl)phenoxy)-[6-^3^H]-phenoxy) propanoic acid ([6-^3^H-phenoxy]-AZ12204657) (Fig. 1b)

(S)-2-(4-chloro-2-(2-chloro-4-(methylsulfonyl)phenoxy)-6-iodophenoxy) propanoic acid (3.1 mg, 5.8 μmol) was dissolved in ethanol (1 mL), and 10% palladium on carbon (2.9 mg, 27. μmol) and triethylamine (10 μl, 71. μmol) were added. The mixture was placed on a RC Tritec Tritium Manifold and the solution degassed by 3 freeze/thaw cycles. Tritium gas (84 GBq, 2.3 Ci) was introduced into the reaction flask and the reaction stirred for 2.25 h at room temperature. The reaction mixture was concentrated; EtOH (0.5 mL) added and concentrated (× 2). The crude product (3.1 GBq) was purified by preparative HPLC (Waters Xbridge C18 100 × 19 mm, 5 μm), using decreasingly polar mixtures of water (containing 0.2% trifluoroacetic acid) and acetonitrile as eluents. Fractions containing the desired product were combined, concentrated, and reconstituted into EtOH to afford 2.2 GBq of [6-^3^H-phenoxy]-AZ12204657. The product had a radiochemical purity of 99% (Waters Xbridge C18, 2.5 μm 4.6 × 75 mm, 5 to 95% MeCN-0.2% formic acid buffered with ammonia to pH 3 over 25 min). The molar activity was determined by mass spectrometry and was found to be 597 kBq/nmol. ^3^H (^1^H decoupled) NMR (533 MHz, *d*_6_-DMSO) δ 7.35 (s); LCMS (ES-) *m/z*: 403 (48%), 404 (10%), 405 (100%), 406 (22%), 407 (60%), 408 (15%).

### In vitro ligand binding assays

The PET radioligand candidate was selected from a chemical series of potent GPR44 antagonists with prospect for labeling with carbon-11 methylation. The potency of AZ12204657 at human GPR44 was determined using in vitro ligand binding assays and membranes of HEK293 cells transfected with human recombinant GPR44 and Gα16, and using a human insulin producing beta cell line (EndoC-βH1) [9].

HEK cells expressing recombinant human GPR44/Gα16 were routinely maintained in Dulbecco’s Modified Eagle’s Medium (DMEM) containing 10% Fetal Bovine Serum (FBS, HyClone), 1 mg/mL G418 (geneticin), 2 mM l-glutamine and 1% non-essential amino acids. For the preparation of membranes, adherent transfected HEK cells were grown to confluence in two layer tissue culture factories (Fisher, catalog number TKT-170-070E). Maximal levels of receptor expression were induced by addition of 500 mM sodium butyrate for the last 18 h of culture. The adherent cells were washed once with phosphate buffered saline (PBS, 50 mL per cell factory) and detached by the addition of 50 mL per cell factory of ice-cold membrane homogenization buffer [20 mM HEPES (pH 7.4), 0.1 mM dithiothreitol, 1 mM EDTA, 0.1 mM phenyl methyl sulphonyl fluoride and 100 μg/mL bacitracin]. Cells were pelleted by centrifugation at 220×*g* for 10 min at 4 °C, re-suspended in half the original volume of fresh membrane homogenization buffer and disrupted using a Polytron homogenizer for 2 × 20 s bursts keeping the tube in ice at all times. Unbroken cells were removed by centrifugation at 220×*g* for 10 min at 4 °C and the membrane fraction pelleted by centrifugation at 90000×*g* for 30 min at 4 °C. The final pellet was re-suspended in 4 mL of membrane homogenization buffer per cell factory used and the protein content determined. Membranes were stored at − 80 °C in suitable aliquots.

The endogenous ligand for GPR44 is prostaglandin D_2_ (PGD_2_) and therefore the ability of unlabeled AZ12204657 to displace binding of [^3^H]PGD_2_ (molar activity 7104 kBq/nmol) was assayed. HEK cell membranes were coated onto wheat germ agglutinin coated polyvinyltoluene (PVT) scintillation proximity assay (SPA) beads (Perkin Elmer). For coating, membranes were incubated with beads at typically 200 μg membrane protein per mg beads at 4 °C with constant agitation for 3–4 h. The optimum coating concentrations were determined for each batch of membranes. The beads were pelleted by centrifugation (800×*g* for 10 min at 4 °C), washed once with assay buffer (50 mM HEPES pH 7.4 containing 5 mM magnesium chloride) and finally re-suspended in assay buffer at a bead concentration of 10 mg/mL. Unlabeled AZ12204657, dissolved in dimethyl sulphoxide (DMSO), was added as 3× dilution series, starting at 1 μM. The DMSO concentration was normalized to 1% in the final assay volume (50 μL). One micromolar of unlabeled AZ12204657 was used as maximum control and 1% DMSO as minimum control. Five-microliter assay buffer was dispensed into the plate, followed by centrifugation at 200×*g*, 1 min. Twenty microliters of 6.25 nM [^3^H]PGD_2_ (final assay concentration 2.5 nM) and 25 μL membrane saturated SPA beads, both in assay buffer, were added. The assay plate was incubated at room temperature for 2–4 h and counted on a Wallac Microbeta liquid scintillation counter (30 s per well).

The ability of unlabeled AZ12204657 to displace [6-^3^H-phenoxy]-AZ12204657 (molar activity 597 kBq/nmol), was quantified in a radio filtration assay. A 96-well flat bottom non-bind PS plate (Corning) was used as assay plate, and a 96-well Multiscreen HTS + HiFlow FB plate (Millipore) was used as filter plate. The assay was run with HEK membranes at 7 μg/well and 3 nM of [6-^3^H-phenoxy]-AZ12204657. Unlabeled AZ12204657 (in DMSO) was dispensed into the assay plate in a 3× dilution series, starting at 1 μM. The DMSO concentration was normalized to 1% in the final assay volume (200 μL). One micromolar of unlabeled AZ12204657 was used as maximum control and 1% DMSO as minimum control in the assay. Twenty microliters of buffer (50 mM HEPES pH 7.4 containing 5 mM magnesium chloride, 10% BSA, *w*/*v*) was added, followed by 20 μL of 30 nM [6-^3^H-phenoxy]-AZ12204657 and 160 μL of HEK cell membranes. The plate was incubated at room temperature for 3 h with shaking. One hundred seventy microliters of the reaction was transferred to the filter plate followed by four times washing with ice cold PBS. The filter plate was dried prior to addition of scintillation solution. After 30 min of incubation, the plate was counted on a Wallac Microbeta liquid scintillation counter (60 s per well).

The potency of AZ12204657 was also determined using a label-free technique measuring the dynamic mass redistribution (DMR) of cells, a technique that confirms activation of G-protein-coupled receptors [10]. Human EndoC-βH1 cells were plated at a density of 2 × 10^4^ cells/well in 384-well fibronectin-coated Epic biosensor plates (Corning) and cultured at 37 °C 5% CO_2_ for 24 h. On the day of experiment, the cells were washed with assay buffer (1xHBSS, 20 mM HEPES (pH 7.4) and 0.2% BSA) and allowed to equilibrate for 1 h inside the Corning Epic Biosensor at 26 °C. Following equilibration, a 5-min scan was performed to create a baseline read before applying AZ12204657 at a concentration range of 1 × 10^−5^ M to 3.8 × 10^−11^ M diluted in assay buffer containing 150 pM 15(R)-15-methyl Prostaglandin D_2_ (Cayman Chem, Ann Arbor, MI, USA) using a CyBi-Well vario. The real-time measurement of DMR was detected during a 60-min scan. Dose response curves were calculated to determine the IC_50_ value using GraphPad Prism 7.

### In vitro autoradiography of binding in pancreas

Pancreatic biopsies were collected from deceased donors (two non-diabetic individuals and one individual with T2D) as well as from a non-human primate (NHP) (cynomolgus monkey). The biopsies were frozen to − 80 °C and processed into 20 μm slices. The use of human tissue was approved by the Uppsala Ethical Review Board (Dnr 2015-401; #2011/473, #Ups 02-577) and tissues obtained from Uppsala Biobank. The use of NHP tissues collected post-mortem was approved by the Animal Research Ethical Committee of the Uppsala Region and was performed according to the Uppsala university guidelines on animal experimentation (UFV 2007/724).

In vitro autoradiography binding studies were performed with both [^11^C]AZ12204657 and [4-^3^H-methyl]-AZ12204657. For all autoradiography assays, pancreatic sections were pre-incubated in 100 mL of 50 mM PBS (pH 7.4) for 10 min. Radioactivity corresponding to approximately 1 nM [^11^C]AZ12204657 or [4-^3^H-methyl]-AZ12204657 was added, and the sections were incubated at RT with each radiotracer for 30 min ([^11^C]AZ12204657) or 3 h ([4-^3^H-methyl]-AZ12204657). Non-displaceable binding was assessed by co-incubation with 20 μM of GPR44 antagonist AZD3825, which is structurally similar to AZ12204657. Each assay was repeated three times with different batches of radioligand.

Following incubation, the pancreatic tissue sections were washed three times for 2 min in 50 mM PBS at 4 °C. The sections were dried and exposed to a digital phosphor-imager screen for 40 min in case of [^11^C]AZ12204657 and 90 h in case of [4-^3^H-methyl]-AZ12204657. A reference of known radioactivity was included to allow for quantification of the results. The screens were scanned using a Cyclone Plus Phosphor imager (Perkin Elmer) at 600 dpi in case of [^11^C]AZ12204657 or a Fujifilm BAS-5000 phosphor imager (Fujifilm, Tokyo, Japan) in case of [4-^3^H-methyl]-AZ12204657. Adjacent pancreatic sections were separately stained for insulin (see details below).

### Immuno-staining for insulin of frozen pancreas sections

Immunofluorescent (IF) staining for insulin was performed on human pancreatic sections adjacent to those used for in vitro autoradiography binding studies. Briefly, sections were stained using Insulin A SC-7839 (Santa Cruz, Dallas, TX, USA; goat-polyclonal 1:1000). The sections were then incubated with secondary antibody Alexa fluor 488 (Invitrogen, Carlsbad, CA, USA; donkey anti-goat; dilution 1:100). Tile scan images were acquired with a Zeiss LSM780 confocal microscope.

Immunohistochemical (IHC) staining for insulin on NHP pancreatic sections was performed by primary antibody A0564 (Agilent, Santa Clara, CA, USA). The stains were developed by the Envision DAB system K4010 using an antirabbit secondary antibody. All sections were counterstained using Mayer’s Hematoxylin. The slides were captured digitally in bright-field mode at × 10/0.45 magnification, using an Axio Imager 2 microscope (Carl Zeiss Microscopy GmbH, Germany) mounted with an Axiocam MRc camera. Images were analyzed in Zen Blue 2.0 (Carl Zeiss Microscopy GmbH, Germany).

### [^11^C]AZ12204657 binding to pancreatic tissue homogenates

Islets of Langerhans and exocrine tissue isolated from a non-diabetic deceased donor was obtained within the Nordic Network for Clinical Islet Transplantation Laboratory in Uppsala, Sweden. The islets (93% pure) and exocrine fractions were separately homogenized in ice-cold 0.32 M sucrose by hand using a Dounce glass homogenizer using a polytron tissue homogenizer (Polytron® PT 3000, Kinematica AG, Littau, Switzerland) in ice-cold 0.32 M sucrose at a concentration of 6 mg/ml and then by hand using a Dounce glass homogenizer. Aliquots of the homogenates were stored at − 80 °C until used.

Two milligrams of homogenized islets of Langerhans and exocrine tissue were separately incubated at RT for 30 min with 1 MBq [^11^C]AZ12204657 (corresponding to 5 nM) in 50 mM TRIS (pH 7.4) in a final incubation volume of 1 mL. Twenty micromolars of AZD3825 was added in a separate assay for determination of non-specific binding. The samples were filtered using a Brandel M-48 cell harvester with Whatman GF/C filter (presoaked with 50 mM TRIS) and washed four times with 3 mL 50 mM TRIS (RT). All samples were performed in triplicates. Filters were measured in a well counter (Uppsala Imanet AB, Uppsala, Sweden).

### Confocal microscopy of GPR44, insulin, and glucagon in human islets of Langerhans

Briefly, human islets were stained with a polyclonal rabbit antibody against GPR44 (HPA014259, dilution 1:10, Atlas Antibodies, Stockholm, Sweden), as well as with rat anti-human insulin (MAB1417, dilution 1:100, R&D Systems, Minneapolis, MN, USA) and Alexa-647-conjugated mouse anti-glucagon (G2654, dilution 1:500, Sigma-Aldrich). Primary antibodies were diluted in UltraAb Diluent (Thermo Fisher Scientific, Fermont, CA, USA) and incubated with the islets for 24 h at 4 °C. The GPR44 antibody was visualized by a secondary antibody conjugated with Alexa-568 and the insulin antibody by a secondary antibody conjugated with Alexa-488 (Dilution 1:500, Invitrogen, Carlsbad, CA, USA). Secondary conjugated antibodies were incubated with the islets for 24 h at 4 °C. The islets were then mounted with Fluoroshield with DAPI (Sigma-Aldrich) and analyzed with Zeiss LSM 510 confocal microscopy (Carl Zeiss, Jena, Germany). A detail of the procedures was described previously [2].

### Statistical analysis

Results are presented as means ± standard deviation. A two-tailed Student’s *t* test was used to test for significance unless otherwise stated (performed in GraphPad Prism 6, San Diego, CA, USA). A star (*) denotes *p* < 0.05; two stars (**) denotes *p* < 0.01.

## Results

### Radiochemistry

The total radiosynthesis time including purification and formulation of [^11^C]AZ12204657 was 30–32 min after end of bombardment (EOB), and the molar activity obtained was 1351 ± 575 GBq/μmol (*n* = 18) at the end of the synthesis (EOS). This one step radiosynthesis was highly reproducible, and it was possible to produce 1484 ± 460 MBq (*n =* 20) of the pure product following irradiation of the target with a beam current of 35 μA for 25–30 min. The radiochemical purity was 98 ± 0.9% (*n = 20*) at EOS and the identity of the radioligand was confirmed by co-injection of the radioligand with an authentic standard of AZ12204657 by radio-HPLC. The formulated solution of [^11^C]AZ12204657 was found to be radiochemically stable for up to 2 h.

To further identify the product, the ion spectrum of the carrier of [^11^C]AZ12204657 was compared to that of the reference compound AZ12204657 using LC-ESI-MS/MS-TOF. The retention time of the reference standard AZ12204657 was 3.08 min, and high-resolution MS/MS of the parent peaks (m/z) 404.8763 (^35^Cl, ^35^Cl), 406.8726 (^35^Cl, ^37^Cl), and 408.8708 (^37^Cl, ^37^Cl) yielded the following fragments (m/z): 358.8848, 360.8804 and 362.8790; 279.9243 and 281.9216; 216.9456 and 218.9430; 154.9807 and 156.9777. The spectrum of the carrier of [^11^C]AZ12204657 was somewhat noisier, because of the lower concentration of analyte in the sample and the parent peak for (^37^Cl, ^37^Cl) could not be detected. Nevertheless the obtained data support confirmation of product identity. The retention time of the formulated product was 3.06 min and high-resolution MS/MS of the observed parent peaks (m/z) 404.8822 (^35^Cl, ^35^Cl) and 406.8776 (^35^Cl, ^37^Cl) yielded a comparable fragmentation pattern as for reference AZ12204657: (m/z): 358.8900 and 360.8874; 279.9207 and 281.9125; 216.9448 and 218.9418; 154.9719 and 156.9837.

### In vitro ligand binding assays

**Fig. 3:**
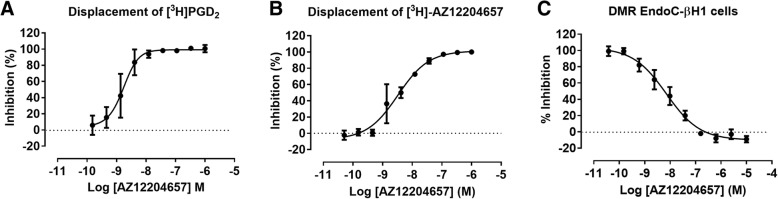
In vitro ligand binding to membranes of HEK293 cells transfected with human recombinant GPR44 and ligand potency at human GPR44 using a clonal beta cell line (EndoC-βH1). [^3^H]ProstaglandinD_2_ (**a**) and [6-^3^H-phenoxy]-AZ12204657 (**b**) binding to human GPR44 was displaced with increasing concentrations of AZ12204657. Results are from two independent experiments with three measurements at each concentration and presented as mean ± SD. The potency of AZ12204657 to inhibit the signal of 15(R)-15-methyl-PGD_2_ in human beta cells measured by the label free DMR assay (**c**). Results are from two independent experiments with two measurements at each concentration and presented as mean ± SD

The in vitro ligand binding assays confirmed AZ12204657 potency at human GPR44. AZ12204657 displaced binding of [^3^H]PGD_2_ from membranes of HEK293 cells transfected with human recombinant GPR44 and Gα16 with a pIC_50_ = 8.6 (Fig. 3a). In the competition assay unlabeled AZ12204657 displaced [6-^3^H-phenoxy]-AZ12204657 binding to GPR44 overexpressing HEK293 cells membranes in a dose response manner with a pIC_50_ = 8.5 (Fig. 3b).

The potency of AZ12204657 in displacing the DMR signal induced by the stable PGD_2_ analog 15(R)-15-methyl-PGD_2_ was determined using the human beta cell line Endo-βH1. Activation of GPR44 with 15(R)-15-methyl-PGD_2_ produced a robust DMR response indicating high expression of GPR44 on the cells. Addition of AZ12204657 dose dependently inhibited the PGD_2_ induced signal with a pIC_50_ = 8.1 (Fig. 3c).

In addition, in a diverse set of in vitro radioligand binding, enzyme and functional assays, covering 81 distinct molecular targets, AZ12204657 was > 1000-fold more potent towards GPR44 than any other target tested (data on file, AstraZeneca).

### [^11^C]AZ12204657 binding in pancreatic tissue

**Fig. 4:**
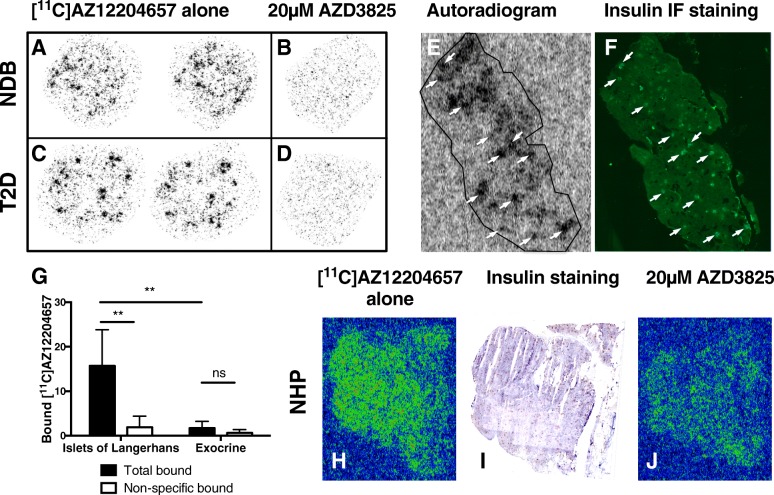
In vitro autoradiography of [^11^C]AZ12204657 reveals focal binding patterns on pancreatic sections from non-diabetic human donors (**a**). The binding could be competed away with an excess of GPR44 antagonist AZD3825 (**b**). Similar GPR44-mediated binding was seen in pancreatic sections from T2D donors (**c**, **d**). The focal binding corresponded to Islets of Langerhans as assessed by immunofluorescent insulin staining (**e**, **f**) (representative results from three independent experiments on human sections with measurements performed in duplicates). The islet specific targeting was further assessed by [^11^C]AZ12204657 binding to homogenates of purified human islets of Langerhans and exocrine tissue preparations (results are from two independent experiments on homogenates with measurements performed in triplicates) (**g**). Binding of [^11^C]AZ12204657 in pancreatic sections from NHP was similarly focal in nature (**h**), consistent with the heterogeneous distribution of islets of Langerhans (**i**), and GPR44-mediated (**j**) (representative results from six independent experiments on NHP sections with measurements performed in singlets or duplicates)

[^11^C]AZ12204657 bound in a heterogeneous pattern in pancreatic sections from non-diabetic individuals (Fig. 4a), and the hot-spots consisted of receptor specific binding as it could be abolished by addition of GPR44 antagonist AZD3825 (Fig. 4b). In pancreas from individuals with T2D, a similar pattern of GPR44-mediated hotspots was apparent (Fig. 4c, d). The hotspots in non-diabetic human pancreas (Fig. 4e) corresponded to insulin-positive islets of Langerhans as shown by IF staining (Fig. 4f).

Binding of 5 nM [^11^C]AZ12204657 in purified islets of Langerhans isolated from human pancreas was GPR44 mediated (*p* < 0.01), and a magnitude stronger than in exocrine tissue from the same pancreas (*p* < 0.01) (Fig. 4g). The weak binding of [^11^C]AZ12204657 in the exocrine preparations was non-specific in nature and could not be consistently competed away.

Pancreatic sections from non-diabetic NHP exhibited a similar heterogeneous binding pattern for [^11^C]AZ12204657 (Fig. 4h), which was co-localized with the distribution of a plethora of smaller insulin-positive islets of Langerhans as assessed by IHC (Fig. 4i). The [^11^C]AZ12204657 binding in NHP pancreas was GPR44 receptor specific (Fig. 4j).

**Fig. 5:**
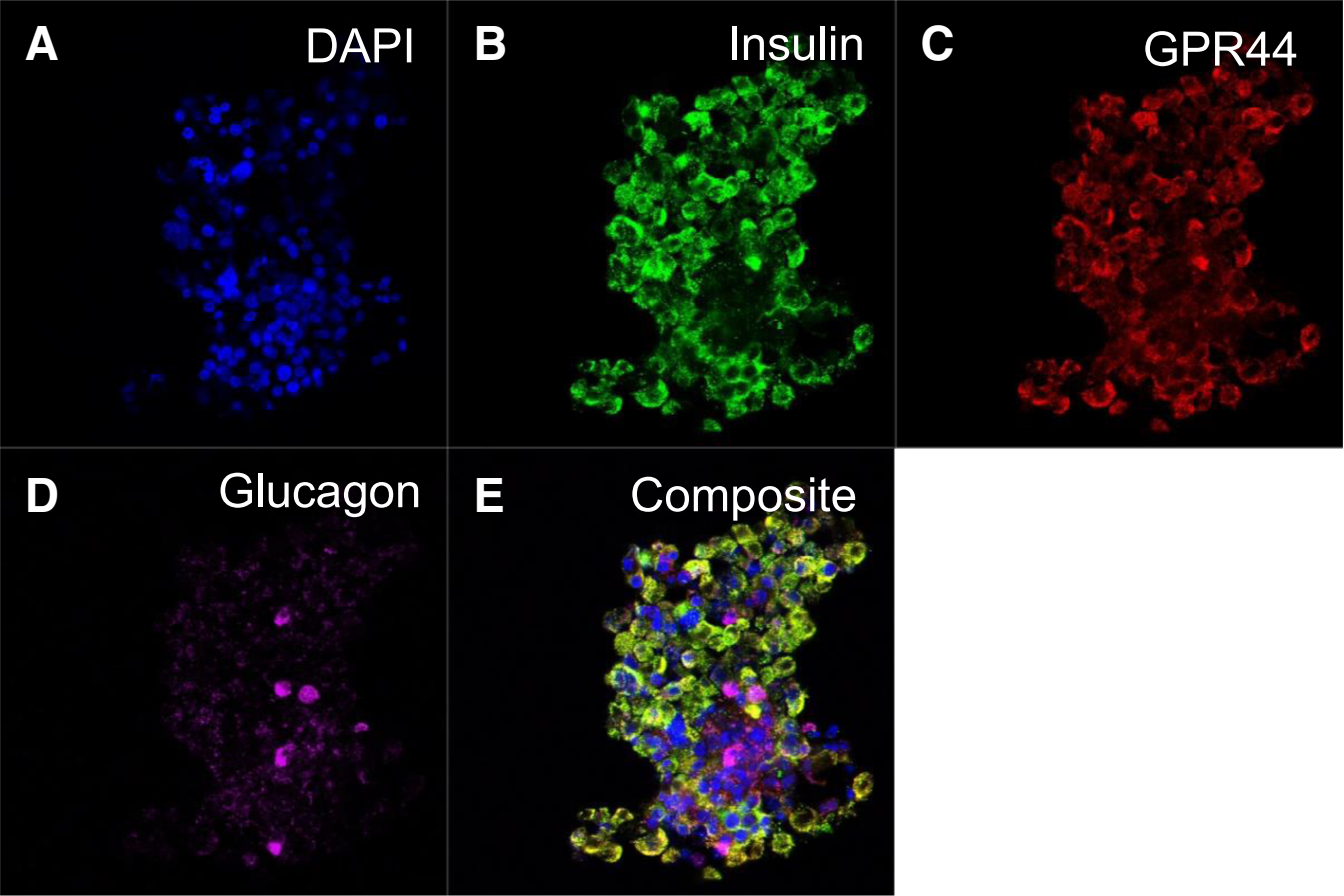
Confocal microscopy of a human islet, showing antibody staining for cell nucleus (blue, **a**), insulin (green, **b**), GPR44 (red, **c**) and Glucagon (purple, **d**). Co-staining between insulin and GPR44 shows up in yellow in the composite image (**e**)

GPR44 receptor distribution was concentrated to the beta cells rather than alpha cells as shown by confocal microscopy of a human islet of Langerhans co-stained for cell nucleus (DAPI, Fig. 5a), insulin (Fig. 5b), GPR44 (Fig. 5c), and glucagon (Fig. 5d). The co-localization between GPR44 and insulin is shown yellow in the composite image (Fig. 5e). Negligible co-staining of GPR44 and glucagon was seen.

### [^3^H]AZ12204657 binding in pancreatic tissue

**Fig. 6:**
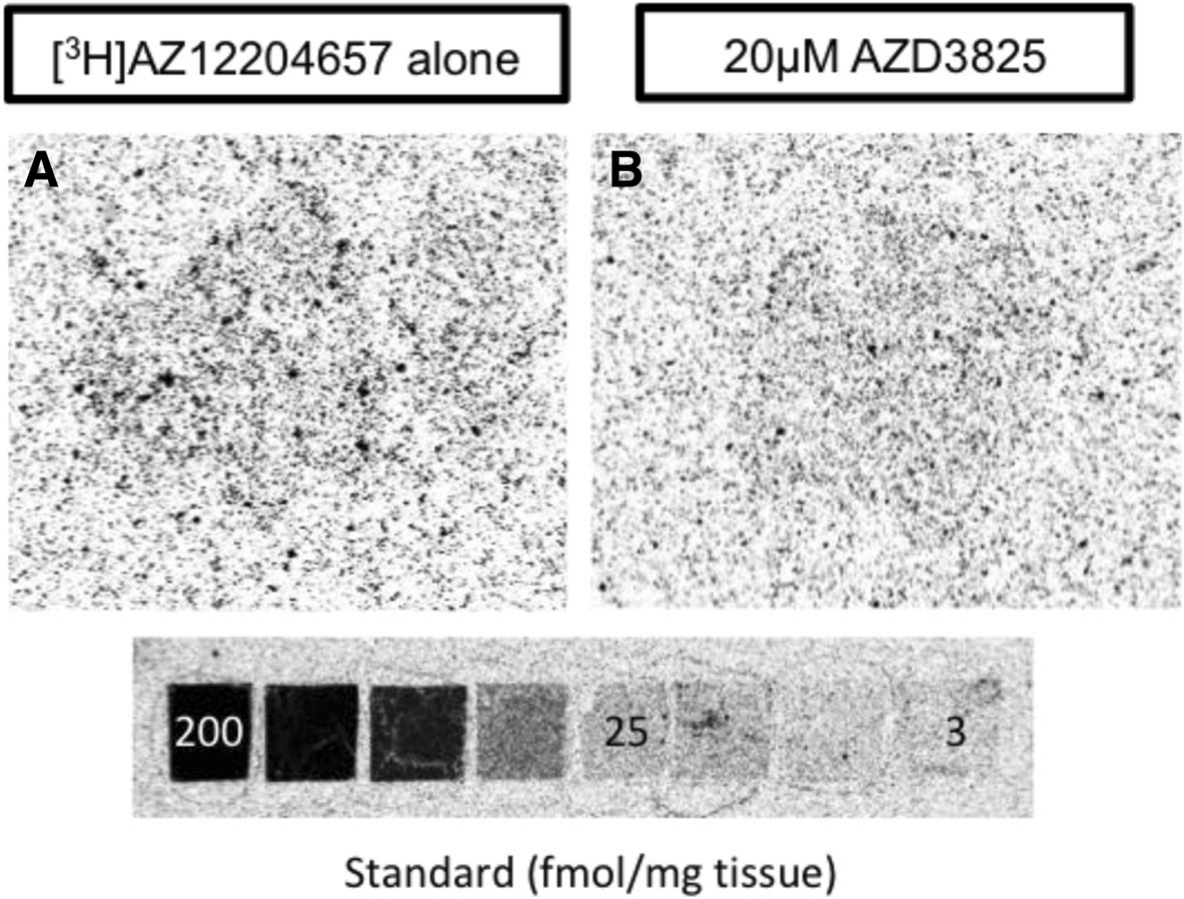
In vitro autoradiography total binding in pancreas by 1 nM [4-^3^H-methyl]-AZ12204657 incubated for 3 h from healthy human subjects (**a**). Non-specific binding is determined in the presence of 20 μM AZD3825 (**b**). The pancreatic binding of tracer is displaceable and concentrated to the islet of Langerhans (results are from two independent experiments with measurements performed in duplicates)

In vitro autoradiography of [4-^3^H-methyl]-AZ12204657 showed heterogeneous pancreatic binding, consistent with the results using [^11^C]AZ12204657 (Fig. 6a). The improved resolution of the radioactive tritium nuclide yielded smaller hotspots, more in line with the size distribution of actual Islets of Langerhans (Fig. 4f) compared to the carbon-11 autoradiograms due to the intrinsic limit of resolution of positron decay. The hotspot binding was almost completely abolished by addition of GPR44 antagonist AZD3825 in excess (Fig. 6b).

## Discussion

Here, the detailed method for carbon-11/tritium radiolabeling, QC, and molar activity determination of novel GPR44 targeting ligand AZ12204657 is presented. In addition to the in vivo evaluation previously reported [7], we here demonstrate further in vitro evidence of the potential of [^11^C]AZ12204657 as a GPR44 binder (e.g., the potency at the receptor by several label-dependent and label-free assays) and as a surrogate marker for beta cell mass (e.g., in vitro autoradiography of NHP and human pancreas and binding to homogenates of pure isolated human islets in comparison with human exocrine tissue).

A rapid and effective one step radiosynthesis of novel GPR44 radioligand [^11^C]AZ12204657 was developed with high MA. Selective *S*-methylation of the precursor (sodium salt of sulfinic acid) was achieved using [^11^C]CH_3_I as alkylating agent to form the sulfone product instead of sulfinate ester product formation via *O*-alkylation. It has been shown previously that alkylation of a sulfinate anion with hard alkylating agents results in predominantly ester formations, whereas soft alkylating agent such as methyl iodide results in mainly formation of sulfone [11] in agreement with the Hard Soft Acid Base (HSAB) principle [12]. The HSAB principle states that hard acids prefer to coordinate to hard bases and soft acids prefer to coordinate to soft bases. In other words, a molecule is extra stable if both the acid and base are hard or if both are soft [13]. In this radiosynthesis (Fig. 1), *methyl carbonium (CH*_*3*_^*+*^*)* is a soft acid and *Iodide (I*^*−*^*)* is a soft base, so *CH*_*3*_^*+*^ coordination to the soft base *Sulphur (S)* of the precursor is preferred over coordination to the hard base *Oxygen (O)*. The position of the radiolabel was confirmed by LC-MS/MS where the product had same retention time and gave an identical fragmentation pattern as that of the reference standard AZ12204657.

Initially, a mobile phase system, MeCN:0.1 M AF without sodium-l-ascorbate, was used for the preparative HPLC purification and the solvent of the collected fraction was removed by evaporation at high temperature. The radiochemical purity of radioligand, [^11^C]AZ12204657, was less than 85% after EOS suggesting radiolysis. Decomposition of PET radiopharmaceuticals is very common via radiolysis, in particular when producing radioligands in high levels of radioactivity and with high molar activity [14] which was the case for [^11^C]AZ12204657. Improved labeling technology and in particular for carbon-11 methylation reactions the development of ^11^C-methyl triflate ([^11^C]CH_3_OTf), a more reactive methylating agent than [^11^C]CH_3_I, has greatly improved the production of ^11^C-labeled radiopharmaceuticals by producing large amount of radioactivity [15, 16]. In the present work, radiolabeling of [^11^C]AZ12204657 was not possible using [^11^C]CH_3_OTf. In initial experiments using [^11^C]CH_3_OTf, a radiolabeled product was formed which did not co-elute with the reference standard, AZ12204657 in radio-HPLC. Though the formed radiolabeled product was not identified, HSAB principle suggests formation of *O*-alkylated sulfonate ester. *Methyl carbonium (CH*_*3*_^*+*^*)* would act as a strong acid when attached to the triflate compared to in the case for iodine and hence prefer to coordinate with the stronger base oxygen with the potential to form the sulfonate ester. The stability of radiopharmaceuticals depends on several other factors such as the structure of the radiolabeled compound, position of radiolabeling as well as the formulated aqueous solution where radicals are generated by interaction between radiation and water. Previous study has shown that addition of appropriate radical scavengers such as ethanol and sodium-l-ascorbate during HPLC purification and the formulation process can improve stability of carbon-11 radiopharmaceuticals [17]. In our case, radiochemical purity of [^11^C]AZ12204657 was improved to > 98% when sodium-l-ascorbate was used in the mobile phase during HPLC purification followed by evaporation of the collected fraction. Impurity of the radioligand was probably due to the concentrated radioactive solution during the evaporation of HPLC solvent. On the other hand, the strong UV absorption of sodium-l-ascorbate sometimes disturbs the quality control of the final product by HPLC. In order to avoid the UV peak of the sodium-l-ascorbate and evaporation of the mobile phase, a method to isolate the product [^11^C]AZ12204657 on a SPE cartridge after HPLC purification was developed allowing removal of sodium-l-ascorbate in the mobile phase. The final product was eluted using ethanol and formulated into PBS which yielded > 98% radiochemical pure compound containing less than 10% ethanol.

Binding experiments using tritiated AZ12204657 or tritiated natural GPR44 ligand, PGD_2_, demonstrated that both radioligands could be fully displaced from human GPR44 by cold AZ12204657 with similar potencies (IC_50_ = 3.2 nM and 2.5 nM, respectively). This together with the data that AZ12204657 could block PGD_2_ signaling in human beta cells suggests specific and reversible binding of the compound to human GPR44.

GPR44 itself has been shown as highly beta cell restricted in the pancreas, by several independent methodologies. Several molecular targets have been suggested as putative imaging biomarkers for pancreatic BCM. The most studied in this regard is currently the glucagon like peptide-1 receptor (GLP-1R), the serotonergic biosynthesis pathway, and the vesicular monoamine transporter 2 (VMAT2) [1]. The development of one or more radiolabeled ligands for each of these targets (for example [^68^Ga]Exendin4, [^11^C]5-Hydroxy-tryptophan and [^18^F]Fluoropropyl-Dihydrotetrabenazine, respectively) have enabled clinical trials assessing their value for measuring human BCM. Despite great advances during the last decade, each of these molecular targets may not be ideal as BCM markers in human, GLP-1R expression in acinar pancreas is highly variable between species [18] and this may account for the background binding of [^68^Ga]Exendin4 seen also in human subjects with T1D; [^11^C]5-Hydroxy-tryptophan is a general endocrine marker and will thus depict both alpha and beta cells in pancreas; and VMAT2 targeting radioligands exhibit a relatively high acinar background binding in most species, including humans, potentially decreasing sensitivity [1]. Thus, a continued search for novel highly beta cell-specific targets is imperative to complement and improve on the abovementioned approaches.

GPR44 was initially selected as a suitable target for beta cell imaging by a proteomic screening in the Human Protein Atlas [19, 20], where the receptor expression in different pancreatic compartments were qualitatively assessed [2]. Importantly, GPR44 was co-localized with insulin (beta cell marker) (Pearson’s correlation *R*_r_ = 0.845) but not glucagon (alpha cell marker) (*R*_r_ = 0.093) [2]. Furthermore, GPR44 was localized on the cellular membrane, readily available for radioligand binding [3]. GPR44 was negative in beta cell deficient islets in long standing T1D, but positive in the rare insulin-positive islets found in the pancreas from acute onset T1D [2]. Transcriptomics of the pancreas corroborated these results; GPR44 mRNA was 26 times higher in human islets of Langerhans compared to exocrine tissue [3]. A tritiated small molecule GPR44 ligand demonstrated an islet-to-exocrine ratio of 20, and a beta cell-to-exocrine ratio of 90 on cellular homogenates [3].

Here, we demonstrate that the first-in-class GPR44 PET tracer [^11^C]AZ12204657 has > 10 times higher receptor specific binding to human islets compared to exocrine preparations. In reality, the islet-to-exocrine ratio of [^11^C]AZ12204657 is likely even higher, since the islet purity was 93% (i.e., some exocrine contamination), and the exocrine preparations following pancreatic digestion and density gradient centrifugation often contain contaminations of islets (approximately 3% islet content in this case). The islet-to-exocrine ratio for [^11^C]AZ12204657 in homogenates is in the range of 20 when taking these contaminations into account. Additionally, the homogenate binding studies were performed at [^11^C]AZ12204657 concentrations (5 nM) above the expected K_d_ of AZ12204657 (IC_50_ = 2.5 nM) due to the MA at the time of the experiments. Thus, at optimized concentration well below the expected K_d_ (as in vivo at microdosing conditions), the specific binding to GPR44 in the islets of Langerhans is likely much higher while the exocrine non-specific binding remains constant.

We further demonstrate that [^11^C]AZ12204657 bind in a GPR44-mediated manner solely to regions positive for insulin on pancreatic sections, i.e., islets of Langerhans. Negligible receptor mediated and non-specific binding was seen in the exocrine pancreas. Confocal microscopy again demonstrated the presence of GPR44 on beta cells but not alpha cells.

The in vitro evaluation was performed on pancreas of human and NHP, firstly due to the strong translational value of this assay and secondly since previous studies in rodent beta cell line INS-1 indicated low binding [3]. Thus, for beta cell imaging purposes, rat does not seem to constitute an ideal animal model for GPR44 and further optimization on rat pancreas or rat derived beta cell lines were not performed.

Despite strong GPR44 expression on individual beta cells, the average density of available receptors in the pancreas as a whole will be low due to the low proportion of islets (2–3%). Due to the intrinsic resolution of PET, individual islets cannot be resolved, and the entire pancreas must be imaged to measure a compound signal proportional to the sum of all pancreatic beta cells (i.e. the BCM) [1]. When the density of available receptors is low, the demand of the radiotracer MA increased, in order to avoid partial saturation of the receptor population (i.e., mass effect or deviation from the tracer concept). Current synthesis of [^11^C]AZ12204657 qualifies the requirements of a tracer designed to measure a receptor with relatively low expression. In clinical settings, with consistent production of this radioligand with MA > 1000 GBq/μmol on average at EOS and in combination with the weight of a human (60–80 kg), we estimate that administration of 300 MBq of [^11^C]AZ12204657 corresponds to a carrier compound’s dosage below 0.01 μg/kg. At such low doses, we expect no mass effect (negligible occupancy) at the GPR44 in the pancreas and thus no appreciable pharmacological action, thereby falling under the microdosing concept.

Carbon-11 is a widely used PET radionuclide potentially allowing for isotopic labeling of carbon containing molecules. In comparison to previously reported islet imaging radioligand [^11^C]5-HTP [21], which is limited in large scale production due to difficult radiolabeling, [^11^C]AZ12204657 offers a simpler labeling chemistry.

In addition, the current combination of small molecule size, short radioactive half-life (20.3 min) and 100% decay by positron emission will contribute to a faster radioactive clearance from blood and tissues, thus yielding lower deposited dose as well as higher resolution PET images compared to previously reported radiometal ligands for beta cell imaging. The low radiation dose of carbon-11 must not be understated: any practical use of an imaging biomarker BCM entails repeated scanning in individuals, either as baseline and follow-up examinations in interventional studies, or several examinations to allow appreciation of the dynamics of BCM during disease progression. Furthermore, carbon-11 labeling enables reliable quantification compared to ^111^In (SPECT) or ^64^Cu (18% decay by positron emission) beta cell-specific tracers [22, 23].

A possible disadvantage of [^11^C]AZ12204657 may be the lipophilicity (CLogP 3.23). Lipophilicity may lead to an affinity for plasma proteins, resulting in tracer accumulation in liver and spleen. This potential issue could be averted by designing ligands to be more hydrophilic upon addition of various amide linkers [24]. Further improvement in image resolution would result from labeling the ligand or a structurally related analog with fluorine-18. Furthermore, the relatively longer half-life of fluorine-18 (109.7 min) would allow for longer chemical reactions, longer dynamic PET scans as well as a potential to distribute the radioligand to PET imaging centers that do not have onsite cyclotron support. The benefits of fluorine-18 of course come with the downside of increasing the absorbed radiation dose thereby potentially limiting the amount of repeated examinations possible in individuals.

## Conclusion

We report the radiolabeling of [^11^C]AZ12204657, a GPR44 selective PET tracer, with high radiochemical purity and high molar activity. [^11^C]AZ12204657 bound to insulin-positive human islets of Langerhans in vitro in a GPR44 mediated manner, but not exocrine pancreas. Confocal microscopy co-staining showed that GPR44 was restricted to beta cells. [^11^C]AZ12204657 is thus a potential PET imaging agent for visualization of BCM.
